# Light makeup decreases receivers’ negative emotional experience

**DOI:** 10.1038/s41598-021-03129-7

**Published:** 2021-12-10

**Authors:** Ling Zhang, Wenfeng Chen, Menghan Liu, Yuxiao Ou, Erjia Xu, Ping Hu

**Affiliations:** Department of Psychology, Renmin University of China, Beijing, 100872 China

**Keywords:** Psychology, Human behaviour

## Abstract

Makeup is widely used in modern society and has a positive effect on perceived attractiveness. However, little is known about the other possible outcomes of makeup use. In this study, we investigated whether makeup enhances a receiver’s emotional experience. Dynamic faces with or without makeup are presented in Experiments 1 and 2. Participants were asked to imagine themselves video chatting with a target person (expresser) with different expressions: neutral, angry, sad, or happy, and then to appraise their own subjective emotional experience. Emotional valence, arousal, and willingness to communicate were also assessed in Experiment 2. The results showed that makeup improved perceived facial attractiveness and increased the willingness to communicate. More importantly, it revealed that wearing makeup could weaken receivers’ negative experiences arising from the angry and sad conditions, which is not the case for the non-makeup condition, but could not affect the happy contagion. Furthermore, incremental changes in the amount of makeup were not accompanied by incremental changes in emotional appraisal (valence and arousal). Overall, we found that makeup may affect emotional contagion and interpersonal communication. Whether the alleviated negative experience due to makeup is adaptive may need further discussion.

## Introduction

Video chat is a widely used communication medium due to its efficiency and convenience. In China, there is a phenomenon wherein individuals attach importance to their own facial attractiveness and attempt to improve their appearance through methods such as the use of the makeup mode during video calls. This led us to the question of whether there is a different emotional experience when a person with or without makeup expresses emotions such as happiness or anger.

Makeup is prevalent in daily life regardless of a person’s appearance and age; its use is encouraged in many situations, such as at work or for appointments. Its most common purpose is to improve facial attractiveness^1–3^, which refers to the positive and joyful emotional experience induced by attractive faces that motivates others to approach the person^4^. Wearing makeup is an intentionally guided strategy of self-presentation^5^, and it is used to cover up facial imperfections and make one appear more charming^6^. People who wear makeup are considered healthier and more confident than those without makeup^7^. Moreover, many benefits associated with natural facial attractiveness can also be experienced with the use of makeup^8,9^. This means that although makeup is artificial, it can achieve psychological consequences similar to those brought on by natural high attractiveness, such as a more positive evaluation of personality traits (e.g., self-confidence, sociability) and the perception of having higher economic or educational status^7,10–13^.

However, it is unknown if this effect also applies to emotional contagion, which refers to the process of transferring an emotion from one individual (expresser) to another (receiver)^14^. The receiver is influenced by the emotion of the expresser, which ultimately results in the receiver’s emotions becoming consistent with those of the expresser^15,16^. Despite the lack of direct evidence, previous research has indicated that individuals with higher attractiveness are more popular in social interactions^17,18^. For example, participants were more willing to participate in games with more attractive partners^19^. By contrast, individuals with lower attractiveness may experience negative treatment and evaluations in their social interactions. They may be subjected to dishonesty^20^, or considered less social or altruistic, or as having lower intelligence^21^. Facial attractiveness not only affects social interactions but also modulates emotional perception and may directly impact emotional experience^22,23^. Nevertheless, there is insufficient evidence to expound on the relationship between makeup and emotional contagion.

Based on the perspective that improved facial attractiveness is associated with certain benefits, it is reasonable to infer that makeup may facilitate emotional contagion via enhanced willingness for interaction and approachability. Facial attractiveness may have a reward value and lead to individuals experiencing positive feelings^24,25^. For example, highly attractive faces are considered to exhibit more positive expressivity than less attractive faces, such that even attractive faces with neutral expressions are usually rated as having positive expressions^26–28^. However, unattractive faces induce negative emotional responses^24^. When participants were required to observe highly attractive and less attractive faces while facial electromyography was simultaneously recorded, researchers found that less attractive faces triggered greater responses in the levator labialis muscle (associated with the disgust emotion) regardless of age (children or adults), implying that faces with low attractiveness may result in receivers’ negative emotions^24^. However, these studies indicate that natural facial attractiveness has more positively correlated social consequences, while beauty achieved artificially through makeup may not always have the same effect^1,2^. Excessive makeup may signify low morality^29^ and trustworthiness^2^ as well as less restricted sociosexuality^30^, but this is a false signal. It remains unknown whether makeup can modulate emotional experiences.

Furthermore, it remains unclear whether the role of makeup varies with the type of emotion. Evidence has supported that interpersonal interactions are affected by facial attractiveness, emotional expression, and the interaction between these two^22,27^. Highly attractive faces enhance the processing of positive emotions and exhibit more advantages associated with happy faces than angry faces^22^, while less attractive individuals are perceived to have more negative expressivity, although their expressions are actually neutral^27^.

Based on the above, four predictions for the role of makeup in emotional contagion may be made. First, emotional contagion will occur; expressers’ positive (or negative) emotions will evoke participants’ positive (or negative) experiences with or without makeup. Second, makeup will affect the degree of emotional contagion by enhancing the perceived valence of emotional expressions, thereby enhancing positive emotional contagion and weakening negative emotional contagion. Third, we hypothesize the effect of makeup on emotions may be unbalanced. Evidence from related studies has indicated an asymmetrical mutual influence of different emotional expressions and facial attractiveness^22,31,32^, which suggests that wearing makeup may only affect positive or negative emotional contagion. Moreover, for neutral expressions, we propose an open hypothesis. On the one hand, neutral expressions with higher attractiveness are usually considered to have positive valence^27,28^. On the other hand, maybe different from emotional perception—emotional contagion is an interactive process, and if there was no obvious intention to transfer positive or negative emotions to the participants, the emotional experience of the receiver may remain unchanged. Fourth, makeup, which increases facial attractiveness and the positive expressivity of faces, may lead to enhanced willingness for interpersonal approachability.

To examine these predictions, we measured the emotional experiences of participants (receivers), who were shown pictures of emotional expressers with or without makeup. Our main purpose was (1) to examine whether makeup can evoke the receivers’ positive appraisal of facial attractiveness, (2) whether makeup affected emotional contagion, and (3) whether this effect was modulated by emotional categories. In light of findings that facial attractiveness and emotional expressions (the magnitude of facial muscle activities evoked by emotions) may affect perceived emotional valence and arousal^27,31,32^, we maintained the same facial expression before and after makeup to ensure that only the makeup was being manipulated. Meanwhile, given that heavy and excessive makeup may have negative effects^1,2^ and that light makeup is often considered more attractive and suitable^33,34^, we ensured the use of light makeup in the current study over heavy makeup.

In addition, dynamic facial expressions (and not static expressions) were employed for the following reasons. First, dynamic facial expressions are more appropriate and effective than static facial expressions for simulating emotional contagion in real life^35,36^. Second, using dynamic video can make receivers more sensitive towards facial attractiveness, thus more easily invoking emotional responses^37^, even though there is no significant difference in the evaluation of attractiveness between static and dynamic pictures^38^. In particular, the materials included both female and male faces displaying various emotions, both of which were treated according the same make-up standard. However, there may be a difference in the perception of men wearing makeup, as this does not conform to gender stereotypes. Although not mainstream, an emerging phenomenon has been occurring wherein young males in China use light makeup to modify their appearance, especially among those seen on TV. Therefore, male faces were also taken into consideration.

## Experiment 1

### Method

#### Participants

Previous studies have observed reliable effects of facial attractiveness on emotion perception or empathy across tasks with approximately 30 participants^23,39^. Considering the uncertainty of whether a potential makeup effect might be weaker and sample sizes recommended by Simmons et al.^40^ and Brysbaert et al.^41^, we adopted a conservative approach and recruited at least 40 participants per condition. Therefore, 48 university students (16 males, age range 18–30 years, *M* = 21.83 ± 2.70 years, sample C, see Table 1) participated in this experiment and were paid ¥50 for their participation.

**Table 1:** The summary of experiment design, critical analysis and outcomes in Experiments 1 and 2.

Study	Tasks	Sample	Sample size	Independent variables	Dependent variables	Analyses	*F or t*	*df*	*p*	Partial *η2* or Cohen’s *d*
Experiment 1	Evaluation of valence, arousal (only non-makeup)	Sample A	27 (male = 10)	Emotion	Valence	Repeated measures ANOVA	262.06	3.78	***	0.91
				Emotion	Arousal	Repeated measures ANOVA	60.19	3.78	***	0.7
	Evaluation of naturalness, attractiveness	Sample B	23 (male = 9)	Emotion*Treatment	Naturalness	Repeated measures ANOVA	0.19	3.66	ns	0.01
				Treatment	Attractiveness (only neutral)	Paired sample t-test (two-tailed)	5.391	22	***	1.108
	Emotional contagion task	Sample C	48 (male = 16)	Emotion*Treatment	Emotional experience	Repeated measures ANOVA	4.803	3.141	**	0.093
Experiment 2	Evaluation of naturalness, attractiveness	Sample B	23 (male = 9)	Emotion*Treatment	Naturalness	Repeated measures ANOVA	1.476	3.66	ns	0.062
				Treatment	Attractiveness (only neutral)	Paired sample t-test(two-tailed)	3.712	22	***	0.774
	Emotional contagion task	Sample D	40 (male = 10)	Emotion*Treatment	Emotional experience	Repeated measures ANOVA	3.062	3.117	*	0.071
	Evaluation of valence, arousal	Sample D	40 (male = 10, one was excluded)	Emotion	Valence	Repeated measures ANOVA	5.22	3.114	**	0.121
				Emotion	Arousal	Repeated measures ANOVA	4.858	3.114	***	0.113
	Further communication choice	Sample D	40 (male = 10)	Attractiveness	Selection proportion	Pearson product-moment correlation	0.668	*		

(1) Table summarizes the design, analysis, and outcomes of the current study is mainly concerned. (2) Samples A, B, C, and D were used to represent different sources of participants. (3) Samples A and B were mainly recruited to complete the experiments on the operational validity test, including the evaluation of attractiveness, naturalness, and emotional attributes (valence and arousal), except for the evaluation of emotional attributes in Experiment 2; Samples C and D were recruited to accomplish the formal experiments (emotional contagion task). (4) To ensure that the manipulation of emotional materials was effective, Sample A was employed to evaluate the emotional valence and arousal before Experiment 1. Meanwhile, given that the evaluation of material naturalness and attractiveness may lead participants to guess the purpose of the emotional contagion task or pay too much attention to whether the emotional expression is natural, Sample B was recruited to evaluate the naturalness and attractiveness of the materials in Experiment 1. Moreover, to ensure evaluation consistency of naturalness and attractiveness between the two experiments, Sample B was also recruited in Experiment 2. Furthermore, in Experiment 2, we used Sample D for emotional contagion, emotional valence, and arousal, considering the need to calculate the correlation between the two. (5) “*”means *p* < 0.05, “**”means *p* < 0.01, “***”means *p* < .001, “*ns*” means *p* > 0.05.

#### Materials

The emotional expression video clips were selected from the Dynamic FACES database^42^ and comprised clips of 38 young actors (23 males) who each performed four two-second facial expression videos, including neutral, happy, sad, and angry expressions, gradually progressing from a neutral face to the maximum emotion. Reactions to the video, such as emotional valence and arousal, were pre-tested on another group of participants (Sample A, 27 students, 10 males, *M* = 21.37 ± 2.44 years old) to ensure effective selection of emotional materials. The participants were required to rate the videos according to the maximum intensity of emotions, as the emotions expressed in the videos gradually increased in intensity. Descriptive statistics (Sample A) are presented in Table 2. Meanwhile, a repeated-measures ANOVA was conducted on valence and arousal; the specific analysis is presented in Supplementary Analysis 1.1. For valence, happy videos were more positive than neutral videos; sad and angry videos were more negative than neutral videos. For arousal, neutral videos resulted in significantly lower arousal than the other videos; happy and angry videos were similar, but sad videos were relatively less arousing than happy and angry videos. In summary, the results showed the manipulation of the emotional materials to be valid.

**Table 2:** Descriptive statistics of materials in Experiment 1 (*M* and *SD*).

Emotions	Treatments	Naturalness (*SD*)	Valence (*SD*)	Arousal (*SD*)
Angry	Non-makeup	4.8 (0.95)	2.66 (0.58)	5.3 (2)
	Makeup	4.8 (0.80)		
Happy	Non-makeup	4.26 (1.12)	7.32 (0.89)	6.23 (1.64)
	Makeup	4.34 (0.88)		
Neutral	Non-makeup	6.06 (1.09)	4.7 (0.75)	2.08 (1.29)
	Makeup	6.12 (1.03)		
Sad	Non-makeup	4.64 (0.92)	3.01 (0.45)	4.81 (1.71)
	Makeup	4.78 (1.1)		

**Figure 1:**
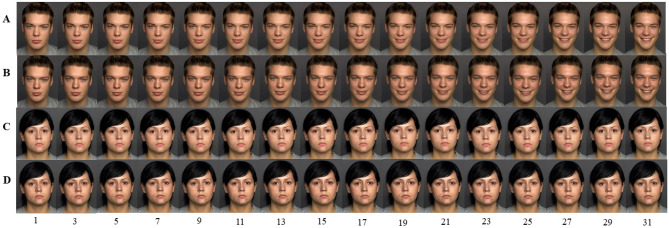
The make-up treatment of emotional videos in Experiment 1. The makeup treatment is shown in Figure. (**A,B**) (happy video) or (**C,D**) (neutral video) were the same emotional videos, (**A,C**) were lightly made up, but (**B,D**) not. The number below indicates the location of this picture in the video (25 frames per second). The original emotional expression video clips were selected from the Dynamic FACES database (No. 066 and No. 140)^42^, and have been permitted to use by its authors.

Subsequently, the faces in these videos were processed with makeup. For doing this, each video clip was broken down into 50 pictures at 25 frames per second. All 50 facial pictures from the same clip were lightly made up following a uniform beauty standard using Photoshop, which included slight whitening and smoothing of the skin and application of pale pink lipstick (see Fig. 1). These treatments were based on the outcomes of makeup applications in real life, such as skin whitening^43^ and smoothing^44^. The edited pictures were then recomposed into a two-second video clip.

The original videos were composed via morphing according to the descriptions in the database. Therefore, both the original and makeup versions may look unnatural. To confirm that both versions matched in terms of how natural they looked and the extent to which makeup enhanced facial attractiveness, another 23 participants (Sample B, nine males, *M* = 19.78 ± 1.88 years old) were asked to rate how natural all the video clips were and how attractive the models were in all neutral video clips—this was done using a nine-point Likert scale, where 1 = *extremely unnatural/unattractive,* 9 = *extremely natural/attractive*. The pre-rating scores are presented in Table 2 (Sample B). Moreover, a repeated-measures ANOVA was conducted on naturalness (see Supplementary Analysis 1.2), which indicated that neutral videos were considered more natural than other videos. However, happy videos were the most unnatural clips, although they did not reach a significant level when compared with sad videos. As previously mentioned, the original videos were edited; therefore, the variations in the teeth may have led to the happy videos being considered the most unnatural. However, applying makeup did not alter the naturalness of videos, which could be demonstrated through a non-significant main effect of treatment and its interaction with emotion. Accordingly, although the manipulation of naturalness was not balanced among emotions, it did not interfere with the effect of makeup and its interaction with emotions on emotional contagion.

#### Procedure

Five minutes before the main task, the participants filled in the Positive and Negative Affect Schedule (PANAS)^45^ to assess their current emotional state.

**Figure 2:**
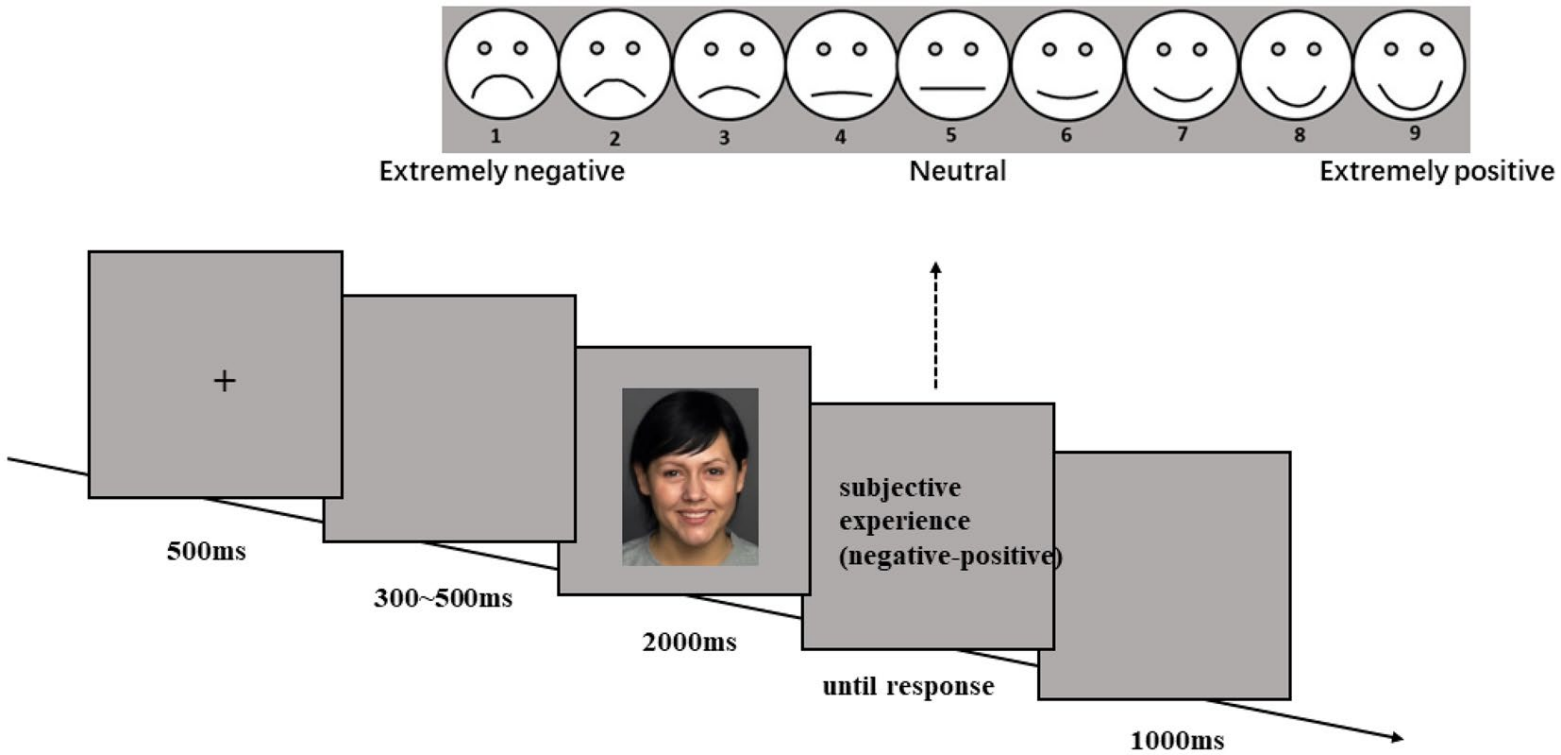
Experimental procedure of Experiment 1. The emotional expression video clips (No. 140) was selected from the dynamic FACES database^42^, and have been permitted to use by its authors.

The participants sat in a quiet room directly facing the center of a screen at 140 cd/m^2^ brightness, where an emotional video of 330 × 430 pixels was presented for 2000 ms. The participants were asked to pretend that they were in a video chat with the person in the video and were informed that “The person in the video may express an emotion to you, and after the video ends, you will be asked to evaluate your own immediate and real emotional experience”, The evaluation was to be made on a nine-point Likert scale, ranging from negative to positive, where 1 = Extremely negative, 5 = Neutral, 9 = Extremely positive. The scenario aimed to simulate the emotional contagion process in a video chat as accurately as possible. The experiment included four blocks with 76 trials each, comprising 19 trials for each of the four emotions. The introduction was reiterated before each block began. The original version of each video did not appear in the same block. The order among blocks and trials was randomized, with a 2-min break between two consecutive blocks (see Fig. 2).

After the experiment, the participants were asked about its purpose. None of them realized that there were makeup and no makeup conditions, and they were unable to determine the experimental goal.

### Results and discussion

#### Manipulation check: the effect of makeup on facial attractiveness (only under neutral conditions)

A paired-sample *t*-test (two-tailed, Sample B) was used to test whether makeup had an effect on attractiveness. As opposed to the non-makeup condition (*M* = 4.32, *SD* = 0.73), the makeup condition (*M* = 4.63, *SD* = 0.75) significantly improved facial attractiveness, *t*_22_ = 5.391, *p* < 0.001, Cohen’s *d* = 1.108.

#### Participants’ emotional state before experiment

The PANAS was measured five minutes before the emotional contagion tasks to confirm the participants’ emotional state before the experiment. The average PANAS scores were as follows: 30.06 (*SD* = 5.95) for positive affect and 17.85 (*SD* = 5.65) for negative affect. A paired-sample* t* test (Sample C) showed that participants were in a moderately positive emotional state, and the positive state scores were higher than those of the negative state before the emotional contagion experiment, *t*_47_ = 10.285, *p* < 0.001, Cohen’s *d* = 2.198.

#### The influence of makeup on emotional contagion

To determine whether makeup affects emotional contagion, we performed a 4 × 2 repeated-measures ANOVA on participants’ self-reported emotional experience (Sample C), with emotion (neutral, angry, happy, sad) and treatment (makeup, non-makeup) as within-participant independent variables. Mauchly’s test of sphericity was adopted to test homogeneity of variance. If Mauchly’s test of sphericity was significant, the Greenhouse–Geisser was employed to correct the results (the same correction method was used for subsequent analysis).

Compared with non-makeup, the makeup condition gave rise to more positive experiences regardless of emotions, with a significant main effect of treatment (*F*_1, 47_ = 13.17, *p* = 0.001, *η*_*p*_^2^ = 0.219). Meanwhile, a significant main effect of emotion was uncovered (*F*_3,141_ = 368.198, *p* < 0.001, *η*_*p*_^2^ = 0.887). Post-hoc tests indicated that regardless of makeup, the emotional experiences of the angry (*MD*_angry-neutral_ =  − 1.776, *SE* = 0.117, *p* < 0.001) and sad expressions (*MD*_sad-neutral_ = − 1.392, *SE* = 0.097, *p* < 0.001) were more negative than the neutral condition, while happy (*MD*_happy-neutral_ = 2.388, *SE* = 0.116, *p* < 0.001) was more positive than the neutral condition. Consistent with the pre-test of emotional valence, angry expressions evoked more negative experiences than sad expressions (*MD*_angry-sad_ = − 0.384, *SE* = 0.045, *p* < 0.001). The results proved that the corresponding emotional experiences of participants were evoked by different emotional expressions.

**Figure 3:**
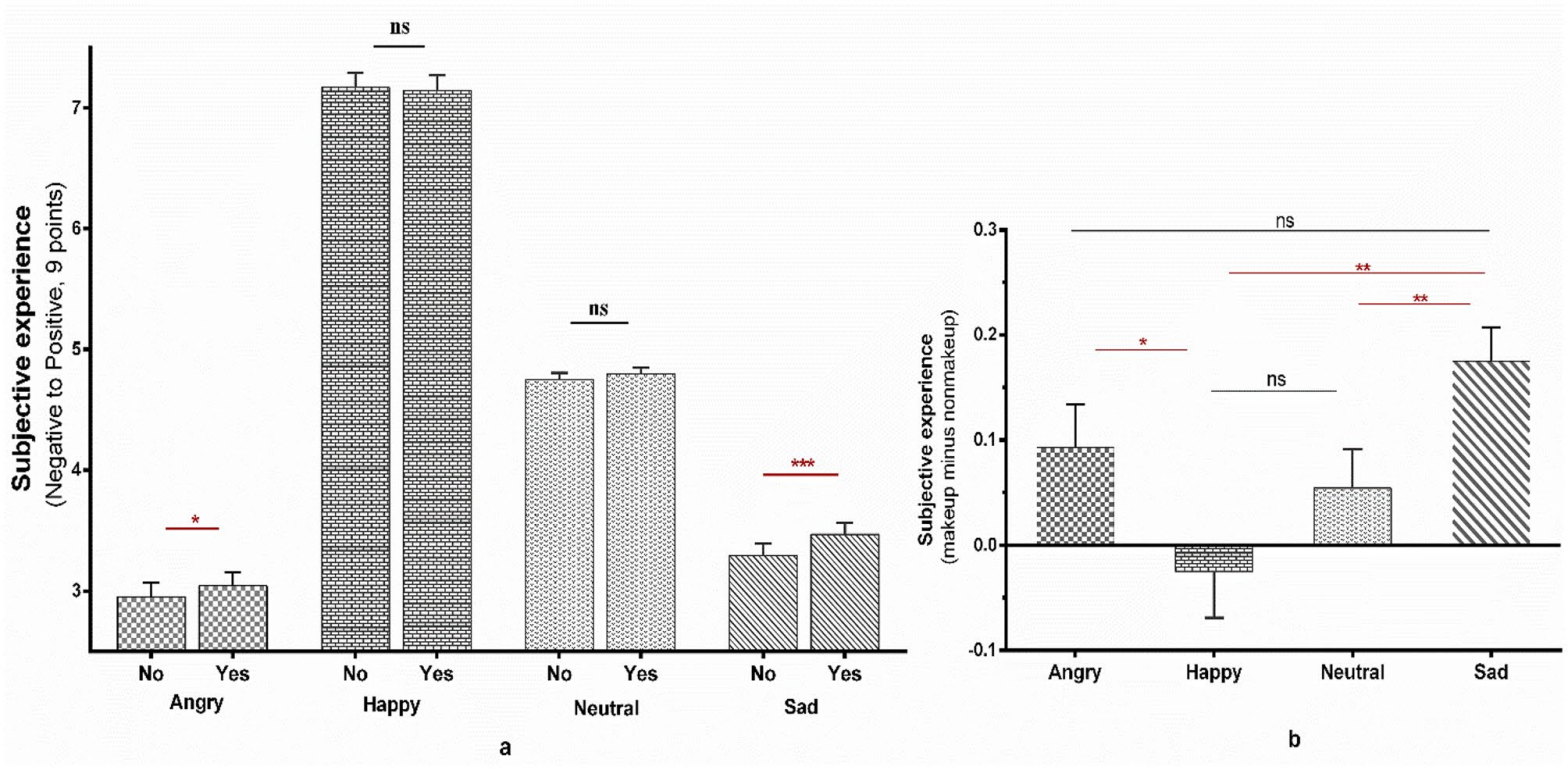
The effect of makeup on emotional contagion in Experiment 1. (**a**) The effect of makeup on different emotional contagion. “Yes” refers to makeup conditions, and “No” indicated non-makeup conditions. (**b**) The differences in increments induced by makeup among emotions. The error bar represents standard error. “*”*p* < 0.05, “**”*p* < 0.01, “***”*p* < 0.001.

Moreover, the interaction between treatment and emotion was significant (*F*_3,141_ = 4.803, *p* = 0.003, *η*_*p*_^2^ = 0.093). A simple effect test revealed that the emotional experiences evoked by faces with makeup were less negative than those evoked by non-makeup faces (angry: *MD* = 0.093, *SE* = 0.041, *p* = 0.027; sad: *MD* = 0.175, *SE* = 0.032, *p* < 0.001). However, this was not the case for the happy and neutral conditions (happy: *MD* = 0.025, *SE* = 0.044, *p* = 0.569; neutral: *MD* = 0.055, *SE* = 0.037, *p* = 0.141; see Fig. 3a; the difference value of makeup minus non-makeup conditions among different emotions was calculated and compared in Fig. 3b, see Supplementary Analysis 6 for detailed analysis).

Overall, Experiment 1 found that makeup weakened the negative emotional contagion under the angry and sad conditions, while the emotional contagion under the neutral and happy conditions remained unchanged. However, it is noteworthy that the video clips in Experiment 1 were morphed, and the happy videos were considered relatively more unnatural than other emotions, which may have affected the impact of makeup on the emotional contagion of happy expressions.

## Experiment 2

Given the exploratory nature of this study and little direct evidence about the interaction of makeup and emotions in the past, Experiment 2 was conducted to verify the results repeatedly with changed materials and participants. As previously mentioned, the stimuli in Experiment 1 were made using morphing technology, which may cause some expressions to look unnatural and could interfere with the experimental results. Therefore, natural emotional expression videos were presented in Experiment 2. In Experiment 1 (Sample A), only the emotional valence and arousal of non-makeup conditions (including happy, neutral, sad, and angry) were rated before to check whether the manipulation of emotions was workable. However, the effect of makeup on emotional evaluation (valence and arousal) and whether it affected the relationship between makeup and emotional contagion is unknown, although previous studies have pointed out that facial attractiveness may modulate emotional perceptions^23^. Therefore, the participants (Sample D) were also asked to complete the emotional evaluation task, for both the makeup and non-makeup conditions, after the emotional contagion task in Experiment 2. In addition to the emotional evaluation and emotional contagion tasks, an extra choice task about further exchanges was also presented to explore the preference for makeup in interpersonal communication.

### Method

#### Participants

Considering that Experiment 2 also explored the correlation between emotional contagion and emotional evaluation, we adopted G*power 3.1.9.2 to estimate the required sample of participants using one-tails, with moderate correlation *ρ* H1 = 0.4, *α* = 0.05, power (1 − *β* err prob) = 0.8; accordingly, at least 37 participants were required. Moreover, as mentioned in Experiment 1, a sample size reaching at least 40 participants per condition would be a more conservative consideration; therefore, another 40 university students (Sample D, 10 males, *M* = 20.98 ± 2.84 years old) were recruited and paid ¥50 for participating. All participants had normal or corrected vision and had experienced no mental or mood disorders in recent days.

#### Materials

Video clips with the highest emotional intensity were selected from the Amsterdam Dynamic Facial Expression Set–Bath Intensity Variations (ADFES-BIV)^46^. There were a total of 48 video clips made up of 13 actors (six males) performing four two-second facial expressions with different emotions (neutral, happy, sad, angry). The progression of emotional intensity in these videos was recorded naturally, rather than being morphed.

The makeup treatments were the same as in Experiment 1, and the same group of subjects (Sample B) were recruited to rate the naturalness of all the video clips and the attractiveness of all the neutral video clips, to maintain consistency between Experiment 1 and Experiment 2 in the evaluation of naturalness and attractiveness.

The pre-rating scores are shown in Table 3 (Sample B), and a repeated-measures ANOVA on naturalness (Supplementary Analysis 4) showed that the naturalness of the neutral expressions was highest, while the other emotional expressions were similar in scores. Moreover, there was no significant interaction between emotion and treatment, nor a main effect of treatment, which implied that makeup did not affect naturalness.

**Table 3:** Descriptive statistics of “naturalness” in Experiment 2.

Emotions	Treatments	Naturalness (*SD*)
Angry	Non-makeup	5.3 (0.98)
	Makeup	5.48 (1.05)
Happy	Non-makeup	5.43 (1.07)
	Makeup	5.33 (1.18)
Neutral	Non-makeup	5.99 (0.96)
	Makeup	6.11 (0.94)
Sad	Non-makeup	5.35 (0.89)
	Makeup	5.31 (1.01)

#### Procedure

The procedure was identical to that of Experiment 1, except for the following. A total of 96 trials were split into four blocks, with 24 trials in each block. Five minutes after the emotional contagion task, the participants were asked to perform an emotional evaluation task that required them to rate the valence and arousal they experienced after watching all the videos. After the emotional evaluation, the participants were asked about the purpose of the experiment, and no one guessed it. Then, the participants completed a choice task. In the choice task, two images of the same person with a neutral expression were presented simultaneously on the screen: one was the made-up version and the other was the original version. The positions (left or right) of the photos were counterbalanced. The participants were told that they were seeing the same person in two different states, and they were asked to choose which image they would be more willing to communicate with.

### Results and discussion

#### Manipulation check: the effect of makeup on facial attractiveness (only under the neutral condition)

A paired-sample *t*-test (two-tailed, Sample B) was conducted to test the effect of makeup on attractiveness. As opposed to the non-makeup condition (*M* = 4.46, *SD* = 0.759), the makeup condition increased facial attractiveness (*M* = 4.86, *SD* = 0.926), *t*_22_ = 3.712, *p* < 0.001, Cohen’s *d* = 0.774.

#### Participants’ emotional state before experiment

Consistent with Experiment 1, the participants’ positive state (Sample D, *M* = 30.43, *SD* = 6.07) was higher than the negative state (*M* = 15.35, *SD* = 3.76), *t*_39_ = 14.66, *p* < 0.001, Cohen’s *d* = 2.305.

#### The influence of makeup on emotional contagion

As in Experiment 1, a 4 × 2 repeated-measures ANOVA was conducted (Sample D) to explore the effect of makeup on emotional contagion. The main effects of emotion (*F*_3,117_ = 341.767, *p* < 0.001, *η*_*p*_^2^ = 0.898) were significant, with angry *(MD*_angry-neutral_ =  − 1.664, *SE* = 0.135) and sad expressions (*MD*_sad-neutral_ =  − 1.124, *SE* = 0.115) arousing more negative experiences and happy expressions (*MD*_happy-neutral_ = 2.481, *SE* = 0.094) evoking more positive experiences when compared with neutral expressions (all *p*s < 0.001) regardless of makeup. Meanwhile, a significant main effect of treatment was also found (*F*_1,39_ = 14.125, *p* = 0.001, *η*_*p*_^*2*^ = 0.266). The faces wearing makeup significantly induced more positive experiences than non-makeup faces regardless of emotions (*MD*_makeup-nonmakeup_ = 0.134, *SE* = 0.036, *p* = 0.001).

**Figure 4:**
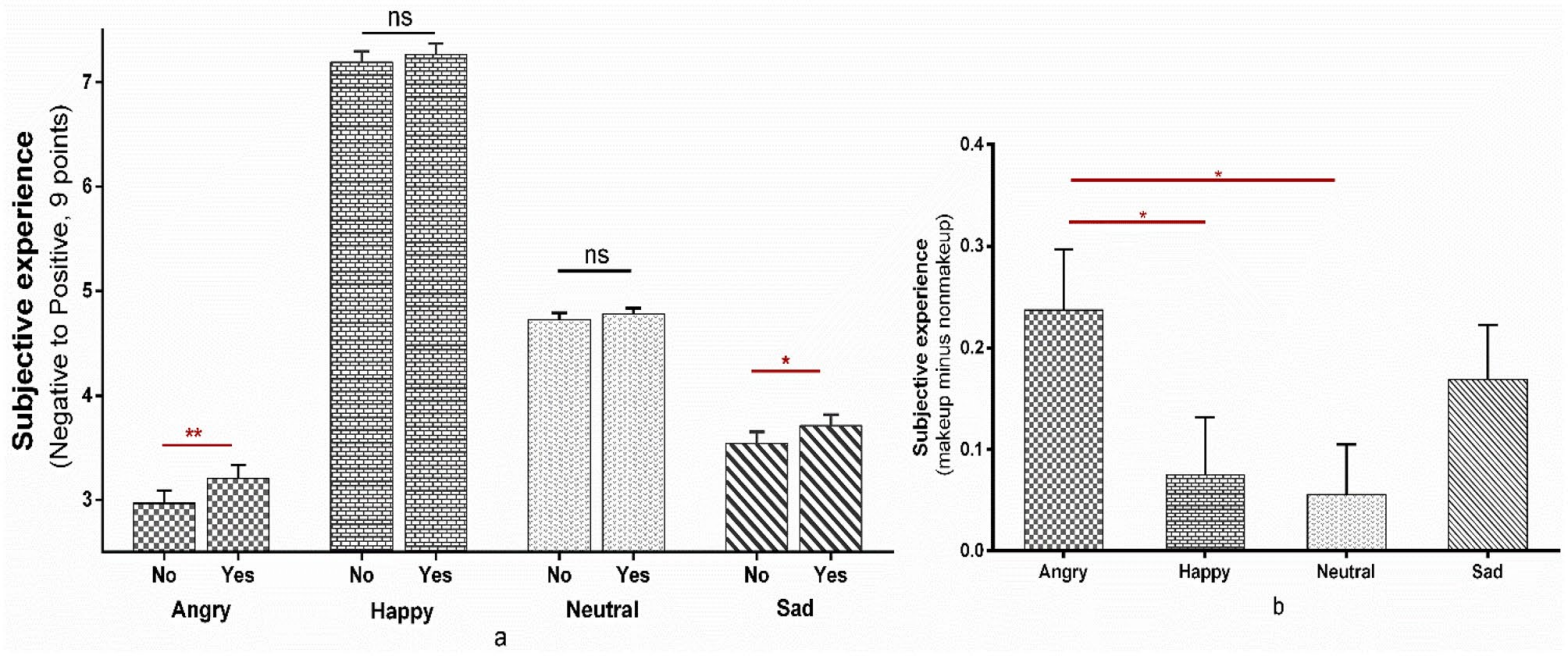
The effect of makeup on emotional contagion in Experiment 2. (**a**) The effect of makeup on different emotional contagion. “Yes” refers to makeup conditions, and “No” refers to non-makeup conditions. (**b**) The differences of increments induced by makeup among emotions. The error bar represents standard error. “*”*p* < 0.05, “**”*p* < 0.01, “***”*p* < 0.001.

Furthermore, the interaction effect between treatment and emotion was significant (*F*_3,117_ = 3.062, *p* = 0.031, *η*_*p*_^2^ = 0.073). A simple effect analysis showed that the emotional experience in the non-makeup condition was significantly negative compared to the makeup condition (angry: *MD* =  − 0.237, *SE* = 0.06, *p* < 0.001; sad: *MD* =  − 0.169, *SE* = 0.053, *p* = 0.003). In contrast, there were no significant differences between the makeup and non-makeup conditions for either the happy or neutral conditions (happy: *F*_1,39_ = 1.703, *p* = 0.2, *η*_*p*_^2^ = 0.042; neutral: *F*_1,39_ = 1.293, *p* = 0.262, *η*_*p*_^2^ = 0.032) (Fig. 4a; the difference value of makeup minus non-makeup conditions among different emotions was calculated and compared in Fig. 4b, see Supplementary Analysis 7 for detailed analysis).

#### The influence of makeup on emotional evaluation

Considering that makeup may change participants’ perceptions and evaluation to match the emotional attributes (valence and arousal) of the expressers, resulting in differences in emotional experiences between the makeup and non-makeup conditions, a repeated-measures ANOVA was conducted with emotion and treatment as within-subject independent variables and valence and arousal as dependent variables. The data of one participant that had been incompletely recorded were excluded (sample D).

For valence, emotion (*F*_3,114_ = 439.12, *p* < 0.001, *η*_*p*_^2^ = 0.92) and treatment (*F*_1,38_ = 11.46, *p* = 0.002, *η*_*p*_^2^ = 0.231) had significant main effects, and their significant interaction (*F*_3,114_ = 5.22, *p* = 0.002, *η*_*p*_^2^ = 0.121) showed that for the angry and sad conditions, the emotional valence under the non-makeup condition was significantly lower than that of the makeup condition (angry: *MD* = − 0.169, *SE* = 0.032, *p* < 0.001; sad: *MD* =  − 0.093, *SE* = 0.045, *p* = 0.046). This suggested that there was a more negative emotional valence for anger and sadness for non-makeup expressers. By contrast, no significant differences were found between the makeup and non-makeup conditions under the happy (*MD* =  − 0.004, *SE* = 0.043, *p* = 0.928) and neutral conditions (*MD* =  − 0.006, *SE* = 0.024, *p* = 0.809).

With respect to arousal, emotion (*F*_3,114_ = 72.24, *p* < 0.001, *η*_*p*_^2^ = 0.655) and treatment (*F*_1,38_ = 5.384, *p* = 0.026, *η*_*p*_^2^ = 0.124) had significant main effects, and the interaction effect (*F*_3,114_ = 4.858, *p* < 0.001, *η*_*p*_^2^ = 0.113) was also significant. A simple effect analysis showed that for the angry and sad expressions, emotional arousal under the non-makeup condition was higher than that under the makeup condition (angry: *MD* = 0.218, *SE* = 0.075, *p* = 0.006; sad: *MD* = 0.181, *SE* = 0.062, *p* = 0.006). However, no significant differences were observed between the makeupand non-makeup conditions under the happy (*MD* =  − 0.037, *SE* = 0.072, *p* = 0.605) and neutral conditions (*MD* = 0.01, *SE* = 0.039, *p* = 0.801).

In summary, we found that wearing makeup increased emotional valence and decreased emotional arousal, which supported our prior hypothesis that applying makeup can change the perception of emotional attributes. However, Song et al.^47^ found that facial attractiveness enhanced the perception of emotional intensity for both neutral and positive emotions rather than anger when using artificial expressions, which is inconsistent with the current study; this may be related to the materials used. The current study used dynamic videos instead of photos and controlled the amplitudes of facial muscle activities under conditions with or without makeup.

#### The relationship between emotional contagion and emotion evaluation

As previously mentioned, we assumed that the different effect of makeup on emotional evaluation may ultimately result in different emotional contagion. Based on the above analysis, we also found a similar result pattern in the simple effect test (after a significant interaction of treatment and emotion) between emotional contagion and evaluation. Therefore, to explore whether the effect of makeup on emotional contagion was related to the change in participants’ perceptions and evaluation of expressers’ emotional expressions caused by makeup, we calculated respectively the differences between the scores of emotional contagion, valence, and arousal under the makeup and non-makeup conditions. We then standardized the increments and used Z-scores to calculate the Pearson product-moment correlation between emotional experience and valence and emotional experience and arousal, respectively, and found that the increments of emotional experience were irrelevant to the increments of emotional valence or arousal regardless of the emotional conditions of angry (*r*_*valence*_ = 0.087, *r*_*arousal*_ = 0.117), neutral (*r*_*valence*_ =  − 0.289, *r*_*arousal*_ = 0.196), happy (*r*_*valence*_ = 0.292, *r*_*arousal*_ =  − 0.065), and sad (*r*_*valence*_ = 0.017, *r*_*arousal*_ =  − 0.05) (all *ps* > 0.05).

We previously presumed that emotional contagion may be affected by emotional evaluation and that the increments of makeup on emotional contagion may be due to the influence of facial attractiveness on emotional appraisal. If the increments of emotional contagion caused by makeup are induced by the increments of emotional evaluation, then the increments brought by makeup should be positively correlated. However, the results did not support the original hypothesis. Perhaps the increments of emotional contagion and of valence or arousal are asynchronous or nonlinear. Another explanation may be that participants’ emotional experiences were affected not only by the emotions expressed by others, but also by factors such as motivation and relationship affinity^48–50^, all of which may result in asynchronous variations.

#### Makeup and further communication choice

In Experiment 2, the participants were tasked with choosing which face they would communicate with more, and it was found that an average of 80.19% of make-up objects were preferred by participants for further communication. The average proportion of participants choosing makeup in the total trials ranged from 38.46% to 100.00%, *SD* = 19.15%. We calculated the differences in the makeup and non-makeup conditions in terms of facial attractiveness and employed an item analysis to calculate the Pearson product-moment correlation between the differences and the proportion of participants who selected faces with high attractiveness (*r* = 0.668, *p* = 0.023). This finding confirmed the common phenomenon that attractive individuals have more opportunity in job applications and interview processes^51,52^.

## General discussion

In brief, Experiments 1 and 2 consistently demonstrated that wearing light makeup can significantly improve perceived facial attractiveness and attenuate negative emotional contagion, as in the angry and sad conditions. The effect of makeup on emotional contagion may be partially mediated by facial attractiveness. We confirmed that perceived facial attractiveness increased when viewing a face wearing makeup through a manipulation test of attractiveness, which was consistent with previous research^8^. The effect of makeup vanished when facial attractiveness was included as a covariate in the analysis of covariance (see Supplementary Analysis 2.5).

Consistent with previous findings, this study indicates that emotions are contagious in social communication. By facial mimicry or social appraisal^53^, the expresser’s emotions can be effectively transmitted to the receiver and evoke the receiver’s similar emotional experience. Emotional contagion is regarded as an adhesive of social relations^54^ and forms one of the bases of empathy^55^, which is an emotional process shared by animals and human beings and has evolutionary significance^16,56–58^.

Furthermore, this study is the first to prove that makeup can affect emotional contagion, similar to its impact on other interpersonal processes^22,27^. In particular, makeup primarily and significantly affected receivers’ negative emotions by reducing the negative emotions felt by them. There are several possible explanations for these results. First, attractive faces brought about by makeup can evoke pleasurable feelings^24^ and may weaken the intensity of negative emotions, thereby resulting in fewer negative experiences. If this is the case, the participants’ emotional experiences would vary according to changes in emotional valence or arousal. However, the analysis of the emotional evaluation in Experiment 2 demonstrated that the increments of emotional experience were irrelevant to both valence and arousal. Therefore, this explanation was inconsistent with our results. Second, the expressers’ facial expressions dynamically and gradually changed from neutral to maximum emotion; therefore, the participants may have appraised facial attractiveness before they detected the negative emotion^9,19,28,59,60^. Consequently, attractive faces may divert more attention from emotional processes, resulting in inadequate processing of negative emotions and subsequently reducing participants’ negative experiences^61^. Third, the preference for attractiveness may have potentially promoted the prosocial motivation of receivers^19,62^. Previous research has pointed out that when more attractive expressers express anger, receivers tend to automatically regulate their negative emotions and impulsive responses to relieve the tense atmosphere, which is regarded as a vital factor in further exchange and cooperation^63^. Our data also indicated that receivers displayed more intent to communicate with individuals wearing makeup.

However, makeup did not affect contagious experiences under the neutral and happy conditions. Evidence on emotional imitation^64,65^ has suggested that positive emotional expressions usually shape a relatively friendly atmosphere; receivers rarely evaluate extra information and automatically imitate it, then rapidly respond with a positive response such as smiling^66^. The effect of makeup on positive emotional contagion may thereby be ignored compared with affinity intention^67^. By contrast, when experiencing negative emotions in which adverse signals are often conveyed, the receivers may appraise the expressers’ personality, intentions, status, and relationships to make appropriate decisions and responses^68,69^. Therefore, negative emotional contagion may be more affected by external information^70^, such as facial attractiveness. Nevertheless, this issue remains controversial^70,71^, and more research is still required to explore and explain why positive contagion is not affected by makeup. Furthermore, for the neutral expressions, because emotional expressers do not obviously transmit positive or negative emotions to receivers in interpersonal contact, no clear affinity or non-affinity motivation was expressed to participants^72^. However, contexts where neutral expressions are expressed can be more formal and serious; therefore, a similar neutral expression may be a more appropriate emotional response in this situation regardless of makeup. However, an analysis based on item (see Supplementary Analysis 2) indicated that made-up faces may cause some positive feelings in neutral conditions and should therefore be further explored.

This study provides evidence for the relationship between makeup and emotional contagion and an available reference for the application scenarios of makeup in social communication, such as election, jury decisions, and service sales. For emotional expressers, individuals can choose to wear cosmetics to adjust facial attractiveness according to their intentions or situation, thereby partially influencing others’ emotional experiences. For example, makeup may be a good choice for concealing emotions when individuals are reluctant to let others feel sadness or pity, such as body makeup at a funeral parlor. On the other hand, if individuals are seeking others’ sympathy or help, wearing beautiful makeup may be futile. Because higher attractiveness is often associated with better survival conditions^9^, it may lead others to misjudge real distress and weaken sympathy and emotional contagion from sad experiences^73,74^. Furthermore, emotional receivers (judges) can realize the attractiveness preference brought about by makeup and then modulate their responses to decrease prejudice or discrimination^75^.

Nevertheless, this study had some limitations that should be considered in future research. First, gender differences were not explored in detail, even though a plethora of research on facial attractiveness has emphasized the importance of gender^8,76,77^. In fact, participants may pay more attention to the opposite gender due to the biological purpose of reproduction^78,79^. However, the evolutionary byproduct explanation supports the idea that attractiveness preference evolved into a cross-gender feature as socialization became more complex and then turned into an indicator of overall quality^80,81^. Although it is not the main concern, our study suggested that the gender of participants and expressers does not obviously change the role of makeup on emotional contagion in Experiment 1 (see Supplementary Analysis 3). Future studies should explore this phenomenon further. Second, the ethnic differences in this study did not account for the participants being Mongolian, as Caucasian faces were used in the videos. The group and cultural differences between the receivers and the expressers in the current study may limit the generalizability of the findings. We are currently collecting and attempting to create a Chinese Facial Expression Video Database and hope that this limitation can be addressed in follow-up research. Third, we performed digital makeup according to the presentation of real makeup, yet there could be some differences between the two. For example, digital makeup may not look as natural as real makeup. Finally, these results are mainly applicable to those wearing light makeup; therefore, whether the findings apply to those wearing heavier makeup requires further discussion.

### Ethics approval and consent to participate

This research was approved by the Renmin University of China research ethics committee. All participants had signed informed consent after being given a complete description of the study and agreed to publish their data publicly. The ethics committee approved this consent procedure and all methods were performed in accordance with the relevant guidelines and regulations.

## Supplementary Information

Supplementary Information.

## Data Availability

Raw data associated with this article can be found in the online version at https://pan.baidu.com/s/10TnMdsoIUAVvzkKhmNh5fQ, extraction code “AB01”. As long as you mentioned our works, you can free access to the data, materials for academic purposes, but not for commercial purposes or for profit. Using the third party material in this article need to contact the appropriate copyright owner for permission.
