# Supplementary material for: Light makeup decreases receivers’ negative emotional experience
Source: Sci Rep. 2021 Dec 10;11:23802. doi: 10.1038/s41598-021-03129-7 (PMC8664826; doi:10.1038/s41598-021-03129-7)
Supplement: Supplementary file 1 — Supplementary Information. [file 41598_2021_3129_MOESM1_ESM.docx]

**Supplementary analysis**

1. **A priori analysis for materials in Experiment 1**

**1.1 The validity of the manipulation: valence and arousal of raw videos (only non-makeup conditions)**

Descriptive statistics are shown in Table 1 (Sample A). Valence and arousal were analyzed through repeated-measures ANOVA to prove the availability of emotional manipulation, with emotion as the within-participant factor. Emotional valence was significantly different among emotions (*F* (3, 78) = 262.06, *p* < .001, *η_p_^2^* = 0.91). Post-hoc tests showed that happy videos were evaluated more positively than neutral videos (*p* < .001), neutral videos were more positive than sad and angry videos (all *ps* < .001), and angry videos were evaluated more negatively than sad videos (*p* < .001). Similarly, the levels of emotional arousal were also significantly different among emotions (*F* (3, 78) = 60.19, *p* < .001, *η_p_^2^* = 0.70). Neutral videos resulted in significantly lower arousal than the other videos (all *ps* < .001); sad videos were significantly less arousing than angry and happy videos (all *ps* < .01), and happy videos and angry videos were similar in terms of arousal (*p =* .176). In summary, the results showed that the manipulation of emotional valence and arousal was valid.

**1.2 The validity of manipulation: naturalness**

The pre-rating scores are presented in Table 1 (Sample B). Because the facial expressions were formed using morph technology, their naturalness could affect the participants’ emotional experiences and confuse the experimental results; therefore, the operational validity of naturalness was tested. A repeated-measures ANOVA was applied to check whether there were differences in naturalness between different emotions, with emotion (neutral, angry, happy, sad) and treatment (makeup, non-makeup) as within-participant variables and the naturalness score as the dependent variable. Only emotion had a significant effect on naturalness (*F* (3, 66) = 19.70, *p* < .001, *η_p_^2^=* 0.47). Post-hoc multiple comparisons showed that neutral videos were considered more natural than other videos (all *ps* < .001), and angry videos were considered more natural than happy videos (*p =* .044), while the naturalness of sad videos was similar to happy (*p =* .111) and angry videos (*p =* .489). As previously mentioned, the original videos were edited, and the variations in teeth may have led to happy videos being considered the most unnatural. However, the main effect of treatment (*F* (1, 22) = 1.25, *p =* .275, *η_p_^2^* = 0.05) and the interaction between emotion and treatment (*F* (3, 66) = 0.19, *p =* .903, *η_p_^2^* = 0.01) were not significant. Therefore, although initial naturalness differed among emotions, applying makeup did not alter the naturalness of the videos.

1. **The influence of makeup on emotional contagion based on the item analysis in Experiment 1**

Considering that the identity of different emotion expressers in the experimental materials may also affect the results, a repeated-measures ANOVA based on item analysis was implemented (Sample C).

First, the main effects of treatment (*F* (1, 37) = 32.796, *p* < .001, *η_p_^2^* = 0.47) and emotion (*F* (3, 111) = 3483.96, *p* < .001, *η_p_^2^* = 0.989) were significant, and the post-hoc test showed that the emotional experience of makeup was higher than the non-makeup condition across emotions, *MD* = 0.076, *SE* = 0.013, *p* < .001. With or without makeup, the emotional experience of happiness was significantly higher than that of neutral (*MD* = 2.283, *SE* = 0.024), while sad (*MD* =-1.299, *SE* =0.034) and angry (*MD* =-1.699, *SE* =0.05) were significantly lower than neutral, and angry was lower than sad (*MD* = -0.4, *SE* = 0.053) (all *p*s < .001). Moreover, the interaction of treatment and emotion was significant (*F* (3, 111) = 10.332, *p* < .001, *η_p_^2^* = 0.218). A simple effect test revealed that compared with non-makeup, makeup improved participants’ emotional experience for the angry (*MD* = 0.071, *SE* = 0.031), neutral (*MD* = 0.055 *SE* = 0.019), and sad conditions (*MD* =0.196, *SE* = 0.032) (all *p*s < .05) but not for the happy condition (*MD* = 0.019, *SE* = 0.025, *p* = .454). Overall, the results showed that makeup could relieve participants’ negative emotional contagion but had no effect on positive emotional contagion, which was consistent with the results based on the subject analysis; this was not the case for the neutral condition. We found that makeup instead of non-makeup induced a relatively positive experience within the neutral condition based on the item analysis, rather than the subject analysis.

Moreover, in a repeated-measures ANOVA based on an item analysis after controlling for attractiveness as a covariate, we found that emotion and attractiveness had a significant main effect (emotion: *F* (3,219) = 208.684, *p* < .001, *η_p_^2^* = 0.741; attractiveness: *F* (1, 73) = 10.748, *p* = .002, *η_p_^2^* = 0.128) and a significant interaction (*F* (3,219) = 6.583, *p* <.001, *η_p_^2^* = 0.083). We also found that the interaction between treatment and emotion (*F* (3, 219) = 4.141, *p =* .007, *η_p_^2^* = 0.054) and the main effect of treatment (*F* (1, 73) = 4.023, *p* =.049, *η_p_^2^* = .052) remained.

1. **The role of gender in the relationship between makeup and emotional contagion (in Experiment 1)**

A repeated-measures ANOVA was conducted considering that the gender of expressers and participants may modulate the relationship between attractiveness and emotions, with the gender of the participants (PGender) as the between-subject independent variable, emotion, treatment, and the gender of expressers (EGender) as within-subject independent variables, and participants’ emotional experience as the dependent variable (Sample C).

The main effect of treatment (*F* (1,46) =10.97, *p =* .002, *η_p_^2^* = 0.193) indicated that, when compared with non-makeup, makeup induced a relatively positive emotional experience regardless of emotion, gender of participants, or expressers. The main effect of emotion (*F* (3,138) =34.612, *p <* .001, *η_p_^2^* = 0.429) manifested that angry expressions led to the most negative experiences, followed by sad expressions, and these two were significantly more negative than the neutral condition (all *ps* < .001). Moreover, happy expressions aroused a significantly more positive experience than neutral expressions (*p* = .001). On the whole, the corresponding emotions arose when participants were exposed to given emotional expressions, which suggested that emotional contagion occurred. Moreover, the main effect of gender was non-significant (*F* (1, 46) =0.026, *p =* .872, *η_p_^2^* = 0.001). However, there was a significant main effect of EGender (*F* (1, 46) =10.97, *p =* .002, *η_p_^2^* = 0.193), which showed that women (rather than men) in emotional expressers led to relatively more positive experiences, regardless of emotions, makeup, or gender of participants.

No significant four-way interaction (*F* (3, 138) = 1.741, *p =* .161, *η_p_^2^* = 0.036), treatment × gender × gender interaction (*F* (1, 46) = 0.375, *p =* .543, *η_p_^2^* = 0.008), or Emotion × Treatment × PGender interaction (*F* (3, 138) = 0.905, *p =* .441, *η_p_^2^* = 0.019) was found. Therefore, the gender of the participants did not modulate the effects of attractiveness on emotional experience.

However, when exposed to different emotional faces, the gender of expressers may adjust emotional experiences according to wearing makeup or not, as indicated by the three-way interaction of Emotion × Treatment × EGender interaction (*F* (3, 138) = 3.773, *p =* .012, *η_p_^2^* = 0.076). The sample effect test showed that there was only a significant interaction between EGender and Treatment for the happy condition (*F* (1, 47) = 11.327, *p =* .002, *η_p_^2^* = 0.194), despite female expressers inducing more positive experiences than male expressers regardless of makeup (*p*s < .05), but their difference was reduced with makeup as opposed to without, *t* = 3.366, *df* = 47, *p* = .002, Cohen’s *d* = 0.574. Importantly, participants felt a higher happiness experience when exposed to made-up, rather than non-made-up faces, regardless of the gender of emotional expressers (*p*s < .001). Except for the happy condition, there were no significant interactions between EGender and Treatment for the anger (*F* (1, 47) = 1.403, *p =* .242, *η_p_^2^* = 0.029), neutral (*F* (1, 47) = 0.885, *p =* .352, *η_p_^2^* = 0.018), and sad conditions (*F* (1, 47) = 1.333, *p =* .254, *η_p_^2^* = 0.028). Therefore, although the gender of emotional expressers may modulate the relationship between makeup and happy contagion, its effect is mainly reflected in the influence degree, rather than in the influence direction of makeup in the happy condition. In general, regardless of the gender of participants and expressers, there was no obvious change in the influence pattern of makeup on emotional contagion.

1. **A priori analysis for the materials in Experiment 2**

**The validity of manipulation: naturalness**

The pre-rating scores are presented in Table 2 (Sample B). A repeated-measures ANOVA was conducted with emotion (neutral, angry, happy, sad) and treatment (makeup, non-makeup) as within-participant variables and the naturalness scores as the dependent variable to inspect whether the naturalness of materials was similar in different emotional conditions; we found that the treatment of makeup does not change the naturalness of materials. Only emotion was found to have a significant main effect (*F* (3, 66) = 3.853, *p =* .013, *η_p_^2^* = 0.149). Post-hoc tests showed that neutral videos were considered more natural than the other emotion videos (all *ps* < .05). However, the scores were similar for the sad, angry*,* and happy video clips (all *p* > .05). Overall, the naturalness of the neutral expression was the highest, while the other emotional expressions were similar in score. However, the treatment did not have a significant effect (*F* (1, 22) = 0.484, *p =* .494, *η_p_^2^* = 0.022), and there was no significant interaction between emotion and treatment (*F* (3, 66) = 1.476, *p =* .232, *η_p_^2^* = 0.062). Therefore, makeup did not affect naturalness.

1. **The influence of makeup on emotional contagion based on item analysis in Experiment 2**

As in Experiment 1, considering that the identity of different emotion expressers in the experimental materials may also affect the results, a repeated-measures ANOVA based on item analysis was implemented (Sample D).

First, the main effects of treatment (*F* (1, 12) = 16.37, *p* = .002, *η_p_^2^* = 0.577) and emotion (*F* (3, 36) = 240.256, *p* < .001, *η_p_^2^* = 0.952) were significant, and a post-hoc test showed that the emotional experience of makeup was higher than that of the non-makeup condition across emotions (*MD* = 0.134, *SE* = 0.033, *p* = .002). Moreover, regardless of makeup, the emotional experience of happiness was significantly higher than that of neutral (*MD* = 2.481, *SE* = 0.194), while sadness (*MD* =-1.124, *SE* =0.123) and anger (*MD* =-1.663, *SE* =0.12) were significantly lower than neutral, and anger was lower than sadness (*MD* = -0.539, *SE* = 0.125) (all *p*s < .01). Although the interaction of treatment and emotion was not significant (*F* (3, 36) = 2.137, *p* = .113, *η_p_^2^* = 0.151), we also adopted a simple effect test to explore the trend of interaction and found that compared with non-makeup, makeup improved participants’ emotional experience for the angry (*MD* = 0.237, *SE* = 0.077) and sad conditions (*MD* =0.169, *SE* = 0.071) (all *p*s < .05) but not for the happy (*MD* = 0.075, *SE* = 0.038, *p* = .07) or neutral (*MD* = 0.056, *SE* = 0.045) condition.

Furthermore, a covariance analysis based on the item analysis showed that when attractiveness was controlled, there was no significant interaction between treatment and emotion (*F* (3, 63) = 0.556, *p* = .646, *η_p_^2^* = 0.024) and no significant main effect of treatment (*F* (1, 21) = 0.409, *p* =.529, *η_p_^2^* = .017). Only emotion had a significant main effect (*F* (3, 63) = 12.32, *p* < .001, *η_p_^2^* = 0.349). Furthermore, the main effect of attractiveness (*F* (1, 21) =18.433, *p* <.001, *η_p_^2^* = 0.445) and the interaction between attractiveness and emotion was significant (*F* (3, 63) = 3.604, *p* = .018, *η_p_^2^* = 0.135).

1. **The size of the makeup effect between different emotions in Experiment 1**

Because the angry videos were initially found to evoke more negative experiences than the sad videos, we calculated the difference between the makeup and non-makeup conditions and then used the difference scores (increments) as the dependent variables and emotion as the independent variable in a repeated-measures ANOVA to determine which emotions were more susceptible to makeup. A significant main effect of emotion showed that the increments for angry were larger than those for happy (*MD* = 0.118, *SE* = 0.053, *p =* .029). Likewise, the increments of the sad condition were larger than those for the happy (*MD* = 0.2, *SE* = 0.056, *p =* .001) and neutral conditions (*MD* = 0.12, *SE* = 0.042, *p =* .006). Moreover, the increments in the neutral and happy conditions (*MD* = 0.08, *SE* = 0.057, *p =*.166) and angry and sad conditions (*MD* = -0.082, *SE* = 0.059, *p =* .173) were similar, as shown in Figure 3b. In short, compared to the non-makeup condition, putting makeup on the emotional expressers mainly decreased the emotional contagion of sadness and anger in receivers, although this did not affect neutral or happy experiences.

1. **The size of the makeup effect between different emotions in Experiment 2**

Additionally, as in Experiment 1, the difference scores (increments) between the makeup and non-makeup conditions were calculated to determine which emotions were more susceptible to makeup. A repeated-measures ANOVA revealed that emotion had a significant main effect, indicating that there was a larger difference between the makeup and non-makeup conditions for anger than for the happy (*MD* = 0.162, *SE* = 0.069, *p =* .025) and neutral (*MD* = 0.181, *SE* = 0.07, *p =* .014) conditions, and the difference was similar to that of the sad condition (*MD* = 0.067, *SE* = 0.068, *p =* .331). In addition, the differences were equal in the neutral and happy conditions (*MD* = 0.019, *SE* = 0.069, *p =* .783). As expected, these results mostly replicated the outcomes of Experiment 1. However, there was a difference in the effect under the sad condition. Although no statistically significant differences were found between the sad and happy or neutral conditions, there was still an increasing trend in the differences (*MD*_sad-happy_= 0.094, *SE* = 0.059, *p =* .115; *MD*_sad-neutral_ = 0.113, *SE* = 0.073, *p =* .126; Figure 4b). In summary, there was a large difference in anger contagion depending on whether the actor was wearing makeup or not, and this was significantly higher than that for other emotions.
